# Chaos analysis of EEG during isoflurane-induced loss of righting in rats

**DOI:** 10.3389/fnsys.2014.00203

**Published:** 2014-10-16

**Authors:** M. B. MacIver, Brian H. Bland

**Affiliations:** ^1^Neuropharmacology Laboratory, Stanford University School of MedicineStanford, CA, USA; ^2^Department of Psychology and Hotchkiss Brain Institute, University of CalgaryCalgary, AB, Canada

**Keywords:** anesthesia, delta activity, sleep, burst suppression, theta, behavior, cortex, consciousness

## Abstract

It has long been known that electroencephalogram (EEG) signals generate chaotic strange attractors and the shape of these attractors correlate with depth of anesthesia. We applied chaos analysis to frontal cortical and hippocampal micro-EEG signals from implanted microelectrodes (layer 4 and CA1, respectively). Rats were taken to and from loss of righting reflex (LORR) with isoflurane and behavioral measures were compared to attractor shape. Resting EEG signals at LORR differed markedly from awake signals, more similar to slow wave sleep signals, and easily discerned in raw recordings (high amplitude slow waves), and in fast Fourier transform analysis (FFT; increased delta power), in good agreement with previous studies. EEG activation stimulated by turning rats on their side, to test righting, produced signals quite similar to awake resting state EEG signals. That is, the high amplitude slow wave activity changed to low amplitude fast activity that lasted for several seconds, before returning to slow wave activity. This occurred regardless of whether the rat was able to right itself, or not. Testing paw pinch and tail clamp responses produced similar EEG activations, even from deep anesthesia when burst suppression dominated the spontaneous EEG. Chaotic attractor shape was far better at discerning between these awake-like signals, at loss of responses, than was FFT analysis. Comparisons are provided between FFT and chaos analysis of EEG during awake walking, slow wave sleep, and isoflurane-induced effects at several depths of anesthesia. Attractors readily discriminated between natural sleep and isoflurane-induced “delta” activity. Chaotic attractor shapes changed gradually through the transition from awake to LORR, indicating that this was not an on/off like transition, but rather a point along a continuum of brain states.

## Introduction

Analysis of electroencephalogram (EEG) recordings have been used to characterize anesthetic effects at various concentration-related depths, from sedation through loss of recall, loss of consciousness, and full surgical immobility. Volatile anesthetics, barbiturates, and propofol produce a stereotypic pattern of EEG changes, with high amplitude slow wave (delta; 1–3 Hz) activity seen during sedation and loss of consciousness, and transitioning to burst suppression patterns at surgical levels of anesthesia (Clark et al., 1973; MacIver et al., 1996; Pilge et al., 2014). Previous studies have used various quantitative measures based on time-series analysis to characterize these EEG signals (Rampil, 2001). Measures based on Fourier, entropy, coherence, and/or bispectral transforms have proven useful in the design of commercially available anesthetic depth monitors. Unfortunately, these monitors have been shown to achieve accuracies/congruencies of only ~ 85–95%, far below a level that is needed to prevent intraoperative awareness (Niedhart et al., 2006; Hrelec et al., 2010).

It has long been known that EEG signals can generate chaotic strange attractors and that the shape of these attractors correlate with depth of anesthesia (Watt and Hameroff, 1988; Walling and Hicks, 2006). One of these studies compared frequency domain measures [FFT: (Walling and Hicks, 2006)], with chaos analysis of anesthetic-induced changes in EEG signals, but the attractor density was too sparse for detailed analysis. The present study used high quality frontal and hippocampal micro-EEG recordings and high density 3D attractor plots to compare signals associated with isoflurane-induced loss of righting reflex in rats. Loss of righting reflex is a commonly used surrogate endpoint measure in rodents for loss of consciousness in humans (Frank and Jhamandas, 1970).

It is possible that anesthetic-induced changes in EEG signals represent altered states of brain processing produced by an anesthetic. In the present study we have tested the hypothesis that chaos analysis can provide a sensitive measure for isoflurane-induced changes in brain state, especially at the point of loss of consciousness. We hope this can contribute to a better understanding anesthesia and theories of consciousness.

## Materials and methods

### Animals

Animal protocols were approved by the University of Calgary Life Sciences Environmental Animal Care Committee in accordance with guidelines from the Canadian Council on Animal Care. All procedures complied with the National Institute of Health (US) and Society for Neuroscience guidelines for the care and use of research animals, and efforts were made to minimize stress, and discomfort at all stages of handling. Thirteen male Sprague-Dawley rats weighing between 300 and 450 gm were used. Rats were obtained from the Animal Care Facility at the University of Calgary.

### Surgery

Rats were deeply anesthetized with ketamine-xylazine 4:1 1.0 ml/kg and placed in a stereotaxic apparatus and prepared for electrode implantation by leveling to horizontal the plane between bregma and lambda as previously described (Bland et al., 2007). Rats spontaneously breathed and body temperature was maintained using a heating pad. An indifferent electrode consisting of small screw was placed in the skull over the cerebellar cortex to act as a ground. Bipolar twisted pair tungsten microelectrodes (Plastics One, Roanoke VA) with vertical tip separations of ~ 1.0 mm were stereotaxically placed in layer 4 of frontal cortex (3.0 mm AB, 3.0 mm L, and 1.5 mm V) and in the CA1 region of dorsal hippocampus (4.0 mm PB, 2.0 mm L, and 2.4 mm V) to record micro-EEG signals. Animals were allowed to recover for at least a week before being placed in a small recording/anesthesia chamber (24 × 10 × 10 inches) that was continuously flushed with room air in control conditions or oxygen that was used as a carrier gas for the isoflurane vaporizer.

### Recording

For experiments, animals were placed in the recording chamber and attached via Plastic One screw type connectors to fine shielded leads and through a commutator to allow free movement. EEG signals were recorded wideband (0.1 Hz–20 kHz) using Grass Instrument Co. P 511 EEG preamplifiers, and were conditioned (x10 gain and zero DC offset) using a BrownLee model 410 instrumentation amplifier, before being digitized at 20 kHz using a National Instruments USB 6009 A/D connected to a MacBook computer running OS10.7/UNIX and Wavemetrics IgorPro acquisition software. Signals were continuously analyzed by FFT and displayed online, and were stored to disk using IgorPro. At least 20 min of control EEG signals were acquired from each rat, consisting of periods of awake immobility, exploring/walking, and sleep immobility, assessed from behavioral, and FFT observation by one of the authors. Once stable baseline recordings were complete, rats were exposed to various concentrations of isoflurane via an Isotec 3 commercial vaporizer. Isoflurane concentrations in the recording chamber were continuously measured and displayed using a Riken FI-21 agent monitor. For loss of righting experiments, isoflurane was applied for at least 20 min to achieve steady-state at each tested concentration and the righting reflex was assessed every 5 min by gently tilting the recording chamber to roll a rat on it's side. At deeper levels of isoflurane anesthesia, rats were placed on a heating pad to maintain body temperature.

### Data analysis

Behavioral observations were time-stamped to recorded EEG signals for off-line analysis/correlation. EEG signals were further processed and displayed as real component magnitude graphs using IgorPro. For chaos analysis, algorithms provided by Walling and Hicks (2006) were utilized and the results were visualized as point plots using 3D graphics in IgorPro. We used an embedding delay of 0.01 s, as this was found to be the minimal delay needed to produce a spherical attractor for the awake frontal EEG signal, sampled at 20 kHz. We found this delay worked well for EEG samples as short as 2 s, but in the examples shown we used the entire EEG trace shown with each attractor (i.e., 6.0–8.0 s). Chaotic attractors were “flattened” by isoflurane in 2 dimensions and this was best seen in 3D rotations. For this reason, we used 3D plots to show the attractors. Quicktime movies of these 3D rotations are provided as supplemental materials. For the graphs shown in this paper, we used a projection that best showed the maximal flattening for each attractor.

## Results

### EEG signals correlated with behavior

**Figure 1:**
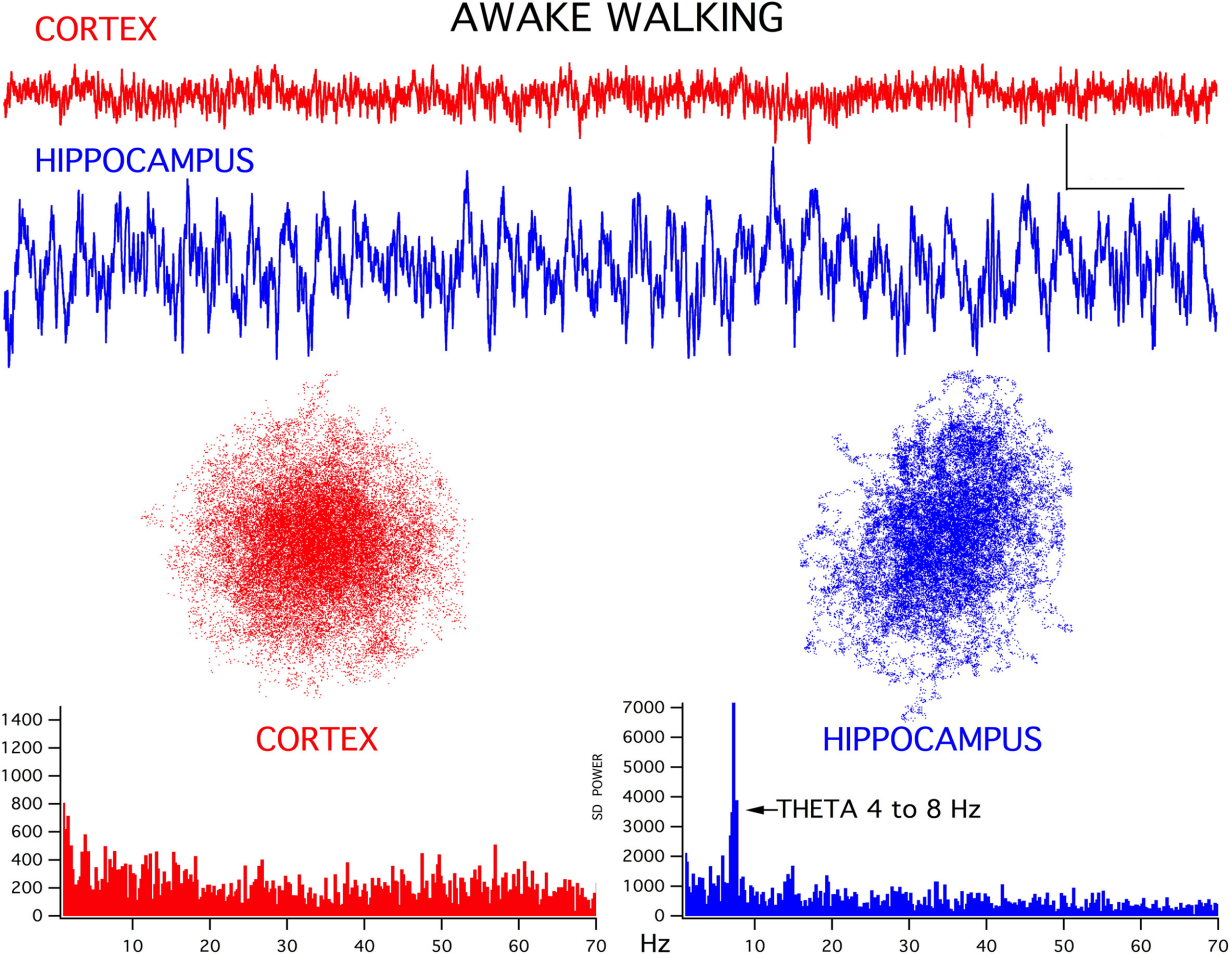
**Representative EEG recordings of frontal cortex (CORTEX) and hippocampal CA1 (HIPPOCAMPUS) signals for simultaneously recorded activity**. During walking/exploring behavior, low amplitude fast activity is seen in cortex and a theta rhythm is generated in hippocampus. Chaotic attractor plots and FFT magnitude graphs, for these two records are shown below the recordings. Calibration bars = 200 μV and 500 ms. Note that the gain of the hippocampal signal was reduced by 1/3 for the analyses, to maintain scaling with respect to cortex.

For both the frontal cortex and hippocampal EEG signals there was a good correlation between ongoing behavior and signal appearance, in agreement with previous studies (Bland and Oddie, 2001). For example, during awake exploring, the frontal cortex generated low amplitude fast activity while the hippocampus produced a theta rhythm (Figure 1). Interestingly, in all thirteen rats, the chaotic attractor associated with this frontal fast activity was spherical, in good agreement with attractors seen in frontal cortical signals recorded from alert humans (Walling and Hicks, 2006). The hippocampal EEG attractor during both theta activity (Figure 1) and large amplitude irregular activity (LIA, not shown) was somewhat flattened compared to cortex. The FFT magnitude graphs associated with these signals showed the typical wideband activity in frontal cortex and a prominent theta peak (4–8 Hz) in the hippocampus (bottom graphs in Figure 1).

### Isoflurane produced a stereotypic change in EEG signals

**Figure 2:**
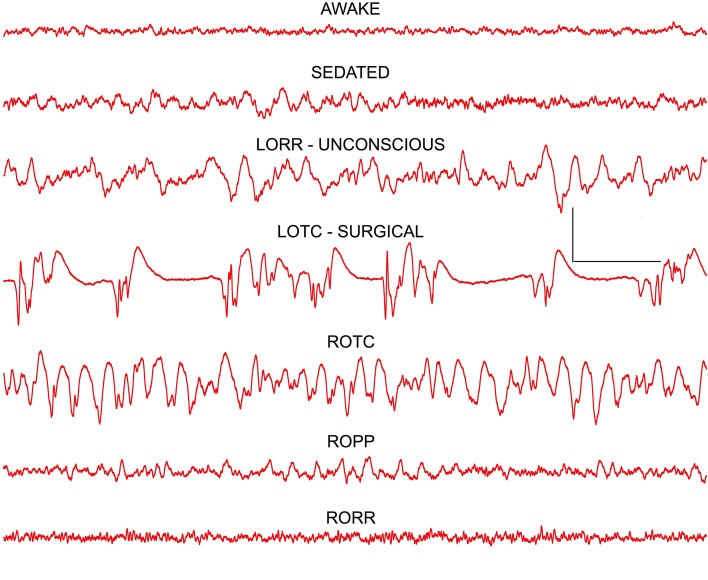
**Frontal cortex EEG recordings showing changes seen at the three anesthetic endpoints (depths) tested: LORR–loss of righting reflex, LOPP—loss of paw pinch, and LOTC—loss of tail clamp**. The dominant effect at loss of consciousness (LORR) was a slowing of the EEG resulting in a high power delta signal that resembled slow wave sleep patterns. At surgical levels of anesthesia (LOTC) burst suppression activity was seen. Note a reverse pattern of changes was seen on recovery as isoflurane concentrations decreased and responses returned (ROTC, ROPP, and RORR). Calibration = 500 μV and 1.0 s.

In agreement with earlier studies in both humans (Buhrer et al., 1992) and rats (MacIver et al., 1996), undergoing thiopental anesthesia, a characteristic pattern of concentration-dependent EEG changes was produced by isoflurane (Figure 2). A very similar pattern was seen in all rats. Low amplitude fast activity seen in awake frontal cortex was replaced by higher amplitude slow wave activity at low concentrations of isoflurane that produced mild sedation. At concentrations of isoflurane that produced loss of righting reflex (LORR; 0.7–0.8 vol %) a further increase in amplitude and slowing of the frontal EEG signal was seen. Loss of tail clamp (LOTC; 1.3–1.5 vol %) response was used as a surrogate endpoint for a surgical plane of anesthesia (White et al., 1974). A characteristic burst suppression EEG pattern was evident in the frontal cortex at LOTC in all rats (Figure 2), although this pattern could change to large amplitude slow wave activity during the course of tail clamp stimulation, and even to an awake-like pattern of low amplitude fast activity in some rats, even though no behavioral response was evident. This type of cortical activation was also seen at lower concentrations of isoflurane during LORR stimulation (see below). Frontal EEG signals rapidly returned to burst suppression patterns within 20 s of removing tail clamp stimulation.

A mirror image of these EEG patterns was seen upon removal of isoflurane from the recording chamber (Figure 2). At return of the tail clamp response (ROTC), high amplitude slow wave activity was evident in cortex. A pattern of activity similar to that seen during mild sedation was evident when rats were able to withdraw their hind leg in response to mild paw pressure (ROPP) and, paradoxically, an awake pattern of low amplitude fast activity was seen for several seconds before rats recovered their righting reflex (RORR in Figure 2).

**Figure 3:**
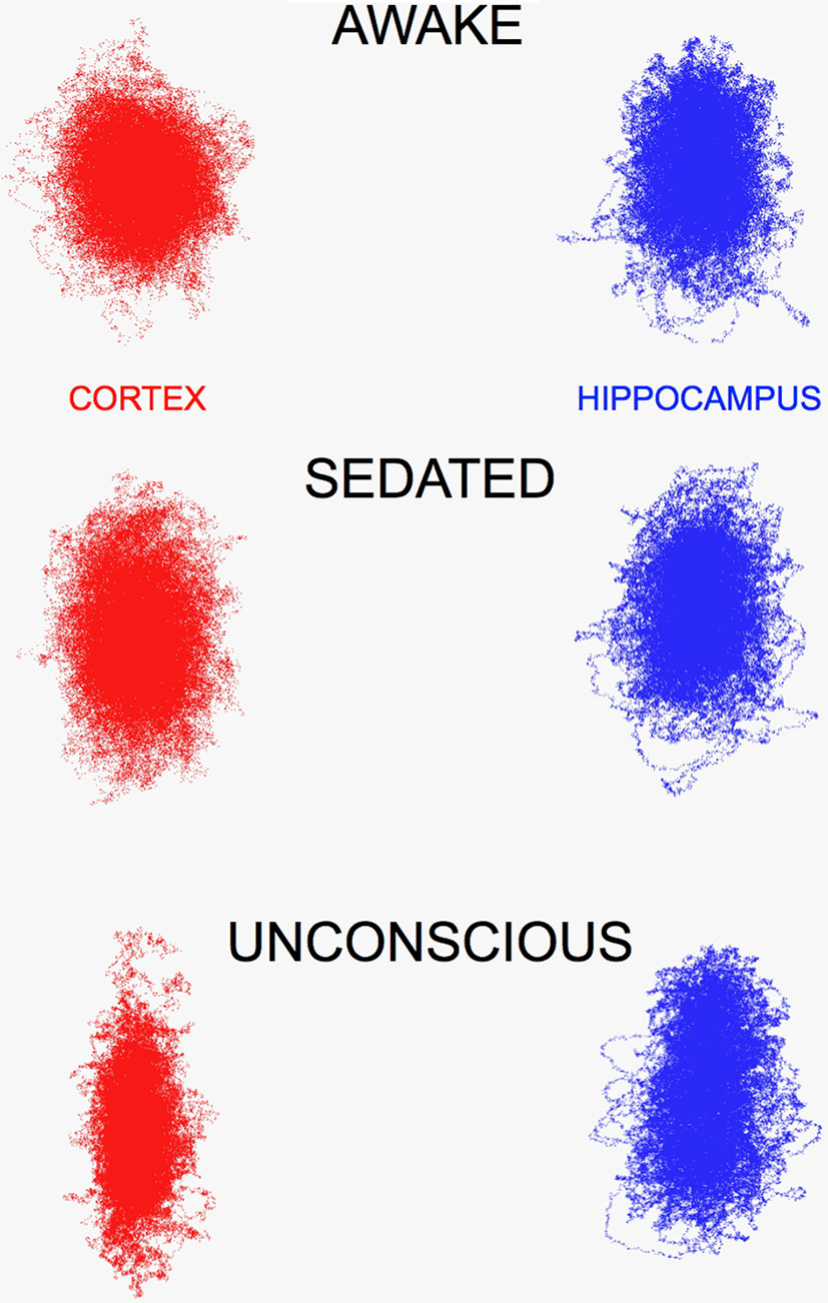
**Chaotic attractors for frontal cortex (CORTEX) flatten in the presence of increasing concentrations of isoflurane (0.5 and 0.72 vol %), however hippocampal attractors remain largely unchanged**. This was evident in recordings too, LIA was the dominant pattern seen in hippocampus, until high concentrations that produced synchronized burst suppression occurred in both brain regions.

While the frontal cortex EEG signals were clearly altered by isoflurane, the hippocampal signals remained largely unchanged, and this was also true for the chaotic attractors (Figure 3), cortical attractors were markedly flattened by isoflurane, but hippocampal attractors remained unchanged.

### Isoflurane-induced slow wave activity was different from slow wave sleep patterns

**Figure 4:**
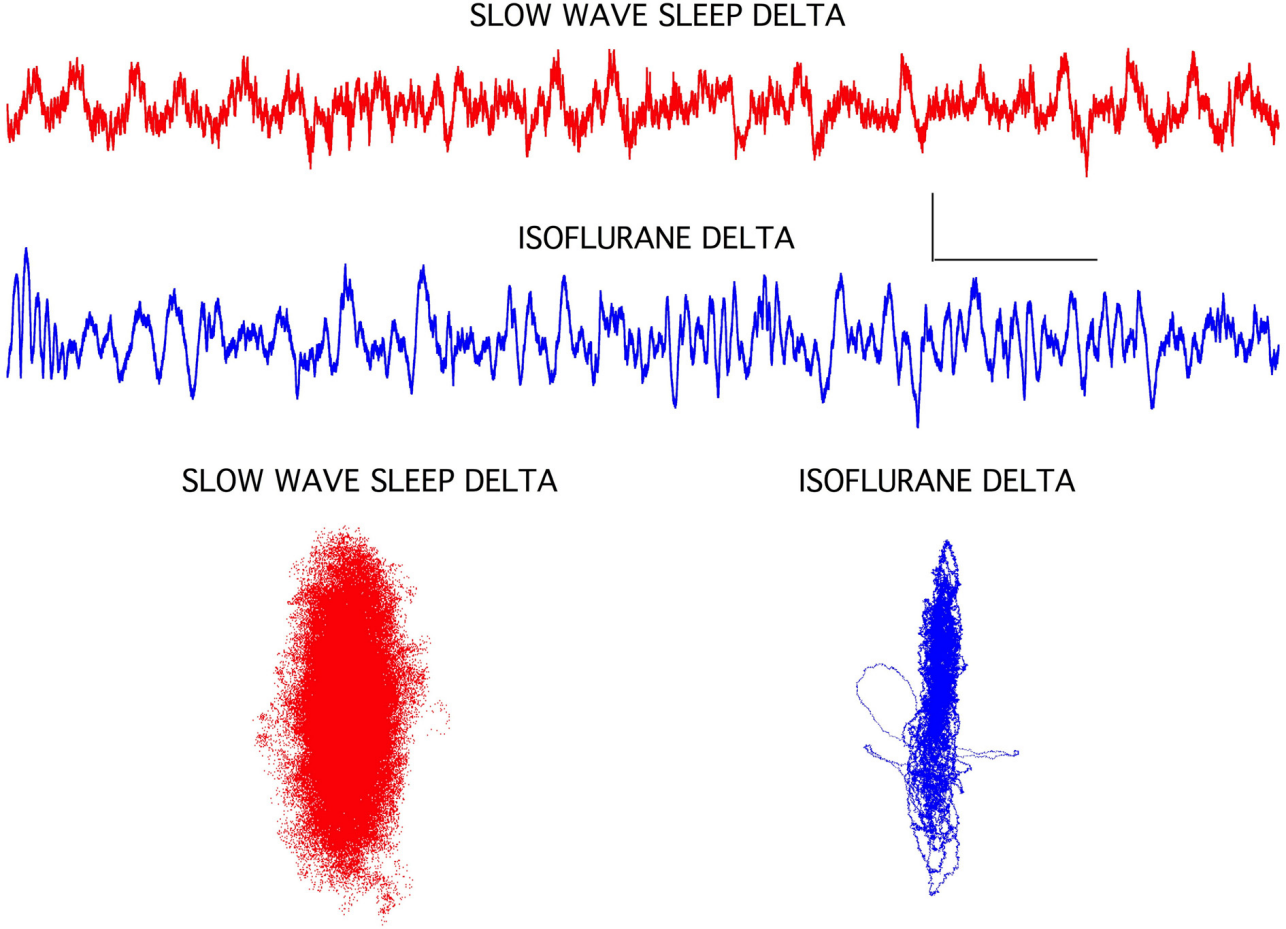
**Slow wave sleep delta EEG activity seen in frontal cortex was associated with a classic sleep posture and with isoflurane-induced loss of righting in rats**. The isoflurane-induced recordings were clearly lacking some high frequency components, but overall amplitude increases and background frequencies were similar. Chaotic attractors were markedly different for these two signals, however, indicating that circuit level differences were associated with these two brain states. Each attractor plots the data for the accompanying EEG recording. Calibration = 300 μV and 1.0 s.

The isoflurane-induced slow wave “delta” activity was quite different from delta activity seen during slow wave sleep in the same rats (Figure 4). Although both EEG signals exhibited similar high amplitudes and a 1–3 Hz dominant frequency, the isoflurane-induced delta activity was notably devoid of higher frequencies seen during sleep. This was clearly evident in the chaotic attractors associated with these two forms of delta activity. Both attractors were flattened compared to the awake condition (Figure 1), but the isoflurane-induced attractor was considerably more flattened and disorganized compared to the sleep attractor (Figure 4).

### Isoflurane-induced LORR

**Figure 5:**
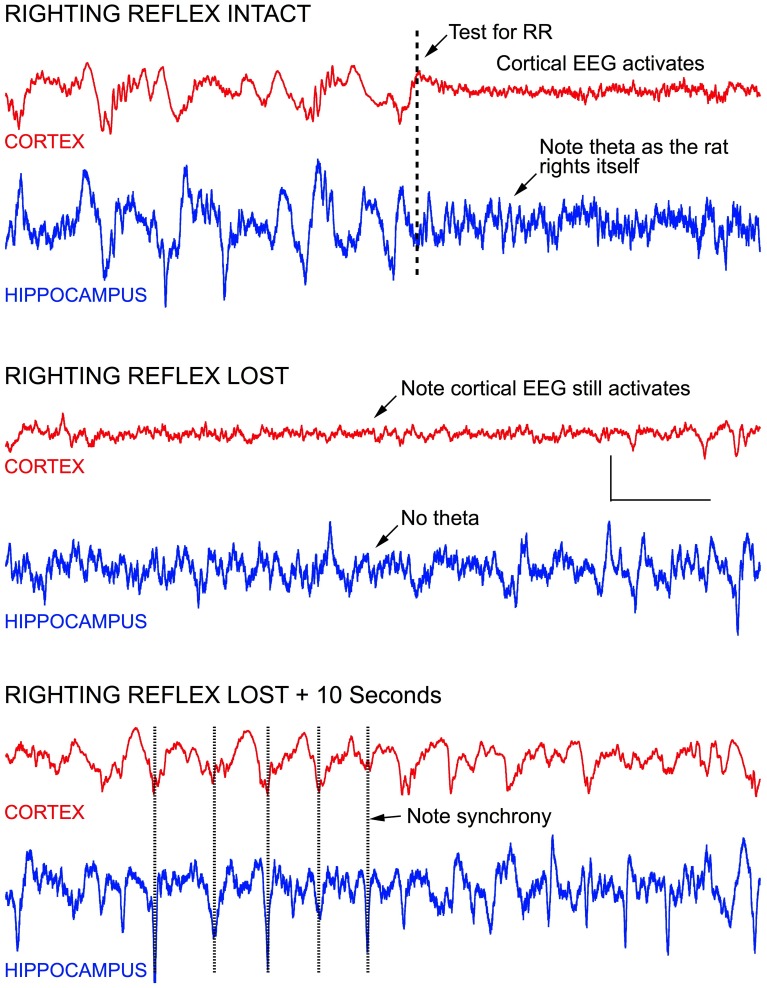
**Stimulating anesthetized rats results in cortical and hippocampal activation. For example, testing the rats righting reflex (arrow in top record) stimulates vestibular, proprioceptive, and other sensory inputs—resulting in an awake-like “activated” EEG signal**. Activation occurred regardless of whether righting was lost or not, and rapidly retuned to a slow wave “delta” pattern seconds after the stimulus onset, regardless of whether the rat righted itself or not. In the middle recordings, the righting reflex was tested 1 s before the start of the recording, and an activated EEG pattern was seen for the entire recording shown (7.0 s), even though the rat failed to right itself. In the lower recording, the EEG is seen to return to slow wave activity within 10 s, and the slow wave activity in cortex appeared to be synchronized with LIA hippocampus. Calibration = 300 μV and 1.0 s.

As mentioned above, testing the righting reflex in rats produced frontal cortical activation, both before and after the reflex was lost. Figure 5 demonstrates this effect. Before testing the reflex the frontal cortex was clearly producing high amplitude slow wave delta activity with sleep spindles seen riding on 2 Hz slow waves, characteristic of isoflurane effects at a concentration of ~ 0.7 vol %. LIA was seen in the hippocampus. When the recording chamber was tilted to test for righting, the frontal signal immediately activated into an awake-like pattern of low amplitude fast activity, and theta appeared in the hippocampus, as the rat righted itself. Five minutes later, at this same concentration of isoflurane, the rat was no longer able to right itself, yet tilting the chamber still resulted in cortical activation, but theta was no longer seen in the hippocampus. Within 10 s the cortical signal returned to high amplitude delta activity and the hippocampus continued to generate LIA (bottom of Figure 5). Over the course of the next 30 min, maintaining this concentration of isoflurane, the rat failed to right on most trials, but was able to right on 2 trials, each separated by failed trails.

**Figure 6:**
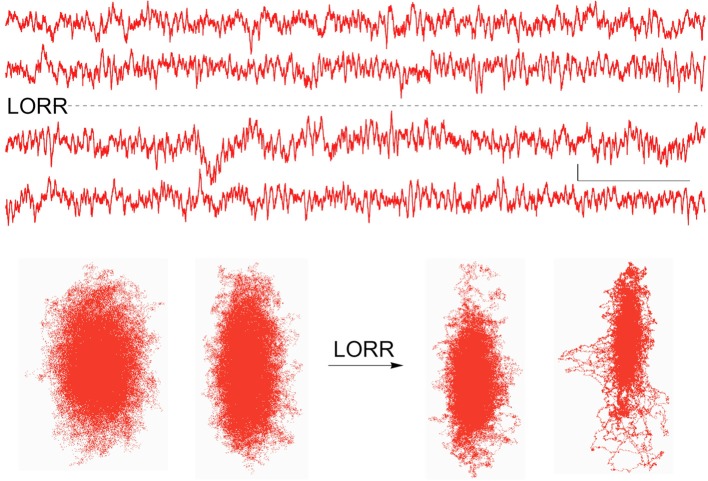
**Activated EEG records from the same rat just before and after isoflurane-induced LORR, together with each records attractor**. Attractor flattening is a sensitive measure of these nearly identical frontal cortical signals. Calibration = 50 μV and 1.0 s.

**Figure 7:**
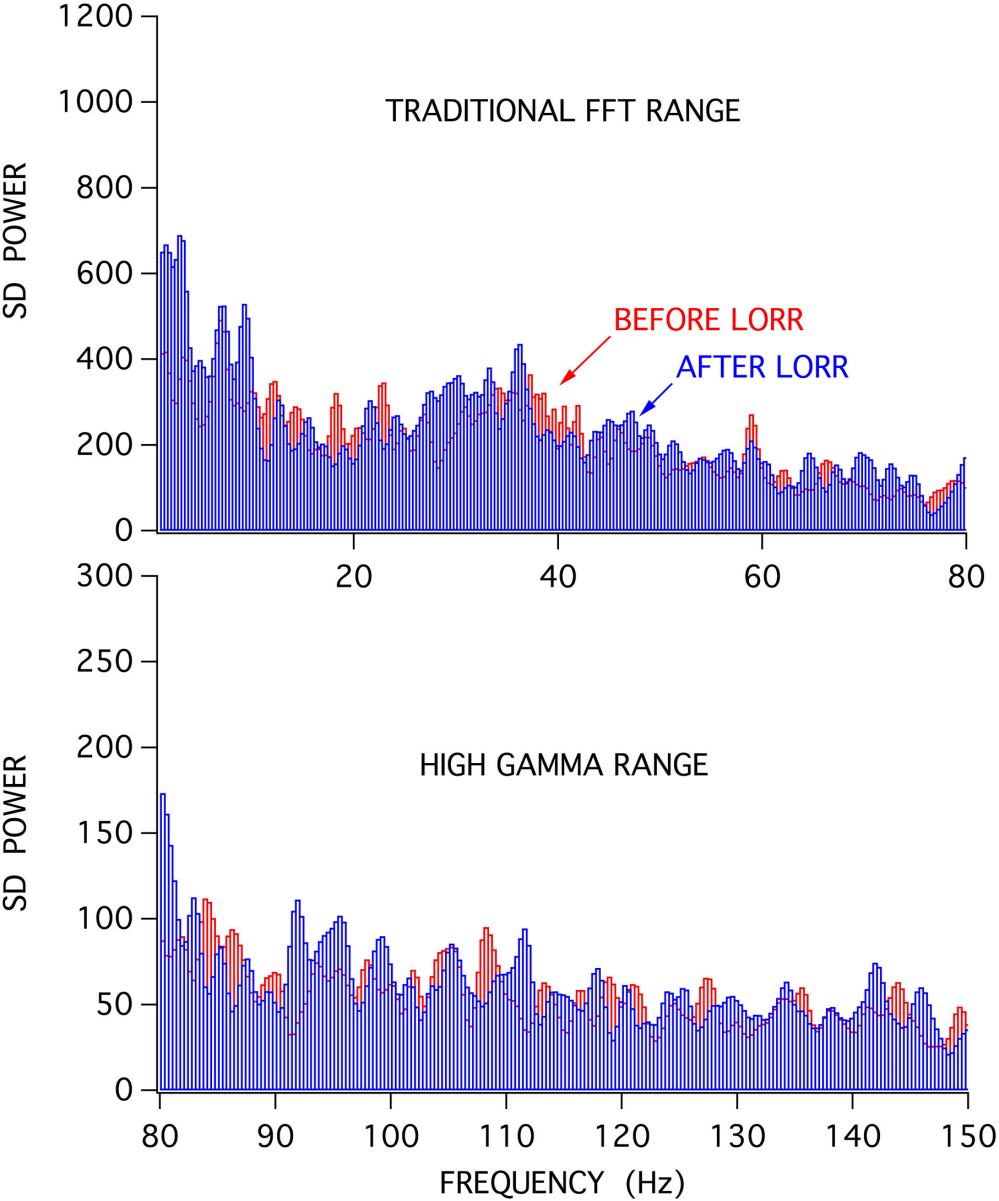
**FFT analysis of activated frontal cortical signals showed relatively little difference in the spectral content of the frontal EEG before (red) and after (blue) LORR**.

When stimulus-induced cortical signals were compared for trials before and after the loss of righting had occurred it was difficult to see any difference in the raw recordings (Figure 6), but there were clear differences seen in the associated attractors. The four recordings shown were for cortical activations before and after LORR, all at the same isoflurane concentration (0.72 vol %) and each separated by 5 min, in a rat that was right on the cusp of loss of righting. In comparison, only small differences were seen in Fourier analysis of these signals. Figure 7 shows FFT magnitude graphs for the two middle traces of Figure 6, just before and just after LORR. As previously reported, there was an increase in delta, little change in gamma (40 Hz), and a slight decrease in high gamma power in the frontal EEG of unconscious rats (Hudetz et al., 2011), but these changes were small compared to changes in attractor shapes (Figure 6).

## Discussion

Isoflurane produced concentration dependent changes in the frontal cortical EEG signal that were similar to patterns seen in other animals, including humans (Li et al., 2013); most notably, a slowing of frequency with an increase in delta power was seen at loss of consciousness. This is similar to patterns produced by other anesthetics in humans and rats (MacIver et al., 1996; Leung et al., 2014; Pilge et al., 2014). Hippocampal EEG signals were largely unaffected by this anesthetic (Figure 3). This contrasts with effects produced by halothane, another volatile anesthetic, that produced a marked hippocampal theta rhythm that persisted even after rats had lost their righting reflex (Bland et al., 2003). The halothane-induced theta rhythm was slower than seen during movement (Perouansky et al., 2010) and likely consists mainly of type 2 (sensory) as opposed to type 1 (movement related) theta (Bland and Oddie, 2001). Halothane also differs from isoflurane in it's lack of burst suppression activity produced in frontal cortex, even at deep surgical levels (Orth et al., 2006; Murrell et al., 2008). Thus, different anesthetics clearly alter higher brain function in an agent, and brain region, specific manner in neocortex and hippocampus, lending support to a multisite agent specific mechanism of anesthetic action (Clark et al., 1973; MacIver and Roth, 1987; Bieda et al., 2009; MacIver, 2014).

This study compared traditional FFT *vs*. chaos analysis of isoflurane-induced changes in EEG signals, and our results suggest that chaos analysis may provide a more sensitive approach. Differences between stimulus-activated signals are considerably easier to discern (Figures 6, 7). Chaos analysis may provide a better approach for the development of monitors for anesthetic depth, as previously suggested (Watt and Hameroff, 1988; Walling and Hicks, 2006). The shape of an EEG driven attractor showed continual flattening in the presence of isoflurane, especially for small changes at the point of loss of conscious behavior. Perhaps a statistical measure of the attractor, like the D_2_ correlation dimension provided by Walling and Hicks (2006), or a simple percent of minimal width measure could provide a wide dynamic range for different levels of consciousness. A wide range is needed to discern stimulus dependent cortical activations produced by testing LORR, LOPP, and LOTC responses, as EEG “awakening” responses are so similar to awake signals (Figure 6). These awakening responses are also seen in patients at surgical planes of anesthesia, in response to particularly painful stimulation, and likely contribute to “awareness” during anesthesia (Hight et al., 2014). A simple display of the frontal attractor in patients could be provided in near real-time on tablet computers and would, at least, provide a sensitive and entertaining view of anesthetic-induced changes in brain state.

Recent studies addressing the similarities between natural sleep and anesthetic-induced slow wave activity have disagreed over shared circuit-level mechanisms (Nelson et al., 2003; Murphy et al., 2011; Zecharia et al., 2012). In some cases a good deal of overlap between anesthesia and sleep was apparent, but in other cases marked differences are seen. Our results with isoflurane indicate that this anesthetic utilizes different mechanisms, or at least additional brain circuit level effects were produced, since the attractor shapes are markedly different between natural sleep and isoflurane-induced slow wave activity. It is likely that any overlap between anesthesia and sleep mechanisms is highly agent and brain region specific.

It remains unclear whether loss of consciousness occurs with an abrupt (on/off) or gradual change in brain state. Our results indicate a gradual effect on attractor shape accompanies the transition through LORR when comparing stimulated “activated” EEG signals, consistent with a gradual return of cortical discharge activities seen on RORR (Vizuete et al., 2014). Of course, spontaneous un-stimulated EEG signals were clearly different at each of the endpoint measures we used (Figure 2), but even the transitions from slow wave sedation through surgical burst suppression occur gradually in the un-stimulated rat as slow wave activity gradually increases in amplitude and gradually breaks up into burst patterns. In humans it appears that unique brain states can exist for different patients on emergence from anesthesia, perhaps related to the degree of painful stimulation on recovery (Hight et al., 2014). This also likely underlies the hysteresis or “neural inertia” evident for anesthetic-induced loss and regaining consciousness, seen in Figure 2 – slow wave activity is seen when rats are not stimulated at LORR, but rats are continuously stimulated by being placed on their sides before RORR, hence the “activated” spontaneous EEG before RORR (Buhrer et al., 1992; Friedman et al., 2010). Perhaps the chaotic attractor measure will provide a wide enough dynamic range to show whether gradual, as opposed to small discrete state changes contribute to loss and regaining of consciousness in humans.

### Conflict of interest statement

The authors declare that the research was conducted in the absence of any commercial or financial relationships that could be construed as a potential conflict of interest.
